# Analysis of the association between MICA gene polymorphisms and schizophrenia

**DOI:** 10.1002/jcla.24721

**Published:** 2022-10-04

**Authors:** Xing Ren, Aiyou Mao, Shumin Tan, Jiaxiu Liu, Xiaobin Wei

**Affiliations:** ^1^ Department of Clinical Laboratory Central South University Xiangya School of Medicine Affiliated Haikou Hospital Haikou China

**Keywords:** genetic polymorphism, MHC, MICA, schizophrenia, susceptibility alleles

## Abstract

**Background:**

The major histocompatibility complex (MHC) has been implicated in schizophrenia. This study aimed to explore the correlation between the major histocompatibility complex class I polypeptide‐related sequence A (MICA) polymorphisms and schizophrenia.

**Methods:**

A total of 220 Han schizophrenia patients, 47 Han healthy controls, 155 Li schizophrenia patients, and 48 Li controls were selected from Hainan Province, China. The diagnosis was made according to the Diagnostic and Statistical Manual of Mental Disorders, 4th edition, criteria. Sequencing‐based‐typing (PCR‐SBT) technology was used for MICA allele typing, and the correlation analyses of MICA gene polymorphism and schizophrenia were performed.

**Results:**

In the Han group, the three allele frequencies of MICA*002:01, MICA*A4, and MICA*A9 in the schizophrenia group were significantly higher than those in the healthy control group, and the differences were statistically significant (*pc* < 0.05; *pc* values were 0.024, 0.030, and 0.031, respectively). Yet, there was no difference in the MICA gene between the schizophrenia group and the healthy controls group in the Li population.

**Conclusion:**

We found MICA*002:01, MICA*A4, and MICA*A9 may be susceptibility alleles for schizophrenia in the Han population, while the MICA allele polymorphism in the Li population is not associated with schizophrenia in Chinese.

## Graphical abstract

This is the first study that explored the role of MICA gene polymorphism in the pathogenesis of schizophrenia. We found MICA*002:01, MICA*A4, and MICA*A9 may be susceptibility genes for schizophrenia in the Han population, while the MICA allele polymorphism in the Li population is not associated with schizophrenia in Chinese.

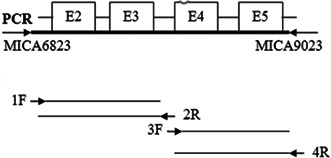

## 1 INTRODUCTION

Schizophrenia is a chronic and disabling mental disorder characterized by hallucinations, delusions, disorganized speech or behavior, and impaired cognitive ability. Most patients present with highly heterogeneous typical symptoms in late adolescence or early adulthood.^1^ A 2016 study showed that schizophrenia's global age‐standardized time‐point prevalence was about 0.28%, with high early mortality.^2^ Moreover, patients with schizophrenia have a high risk of suicide, and their life expectancy is approximately 20 years shorter than in the general population.^3^ Intermittent and long‐term mental problems, chronic symptoms, and even disability often lead to extremely high unemployment among people with schizophrenia, with profound implications for individuals and societies.^4^

The etiology and pathogenesis of schizophrenia are still unclear. Genetics, environment, neurodevelopment, inflammation, and immunity are possible pathogenic factors. It is generally accepted that the interaction of genetics and the environment has an important influence on schizophrenia.^5^, ^6^ Genome‐wide association studies identified a number of risk loci with polymorphisms, and numerous studies have demonstrated the important role of genetics in susceptibility to schizophrenia.^7^ Moreover, epidemiological and animal model evidence suggests that maternal infection is a risk factor for schizophrenia and that maternal immune activation (MIA) alone is sufficient to lead to lifelong neurological and behavioral changes in offspring.^8^ Subsequently, another study pointed out that the cause of MIA in schizophrenia is associated with the production of cytokines and complement proteins in the immune system that affects neural development.^9^ It has been suggested that complement levels are potential peripheral biomarkers in schizophrenia.^10^, ^11^ Inflammation has an important role in pathogenesis and maintenance, and cytokine disturbance is associated with disease staging.^12^ Schizophrenia has also been associated with chronic low‐grade inflammation, while an abnormal immune system has been identified as a risk factor for schizophrenia.^13^ Clinical trials have demonstrated that immunomodulation improves psychiatric symptoms in patients with schizophrenia.^14^

Recent multiple genome‐wide association studies identified a strong genetic association between MHC locus and schizophrenia.^15^ MHC, also known as human leukocyte antigen (HLA), is located on the short arm of chromosome 6 (6p21.3–22.1) and has a high degree of polymorphism and extensive linkage disequilibrium (LD).^16^ The MHC region is divided into class I, class II, class III, and extended class I and II genes. Several MHC‐related single nucleotide polymorphisms in schizophrenia have been previously reported.^17^, ^18^ HLA‐G, a non‐classical MHC class I gene, is associated with the risk of developing schizophrenia and the severity of its clinical symptoms.^19^, ^20^ The major histocompatibility complex class I chain‐associated gene A (MICA) is located at the centromeric terminal of the HLA class I‐associated region, adjacent to HLA‐B, and belongs to the non‐classical HLA class I family. MICA does not present any antigen but acts as a ligand for natural killer (NK) cells, γδ T cells, and αβ CD8+ T cells, which express a common NK cell receptor natural killer group 2D (NKG2D).^21^ MICA protein is absent from most cells but can be induced by infections and oncogenic transformation.^22^ MICA is highly polymorphic. Previous studies have linked MICA to rheumatoid arthritis, ankylosing spondylitis, Behçet's disease, celiac disease, and type 1 diabetes.^23^, ^24^, ^25^, ^26^, ^27^ In addition, some studies have shown a bidirectional association between these autoimmune diseases and an increased risk of schizophrenia.^28^, ^29^

This study aimed to analyze the association between MICA gene polymorphisms and schizophrenia in Han and Li populations in Hainan Province, located at the southernmost tip of China, isolated from the mainland, where the Li and Han nationalities account for more than 98% of the province's population. To the best of our knowledge, this is the first study that explored the role of MICA gene polymorphism in the pathogenesis of schizophrenia.

## 2 MATERIALS AND METHODS

### 2.1 Subjects

A total of 275 schizophrenia patients and 95 healthy controls from Hainan Province were selected. Among them, there were 220 Han schizophrenia patients and 47 Han healthy controls, and 155 Li patients and 48 Li healthy controls. The general clinical information is shown in Table 1. All the enrolled people came from families in Hainan Province, whose paternal and maternal lineages were Han (or Li) for more than three consecutive generations. Enrolled patients were not related. This research protocol has been approved by the Biomedical Ethics Committee of Haikou Hospital Affiliated with Xiangya School of Medicine of Central South University (Haikou People's Hospital) with approval number 2019‐(ethical review)‐087. Informed consent was obtained from all subjects or their guardians.

**TABLE 1:** Clinical information of patients with schizophrenia and healthy controls

Clinical information	Han SZ patients (*n* = 220)	Li SZ patients (*n* = 155)	Han HC (*n* = 47)	Li HC (*n* = 48)
Gender, *n* (%)				
Male	150 (68.2%)	103 (66.5%)	24 (50.9%)	24 (50.9%)
Female	70 (31.8%)	52 (33.5%)	23 (49.1%)	23 (49.1%)
Age (years)	15–77	14–75	12–49	5–56
Onset age (years)				
Average	24.2	26.5	–	–
Family, *n* (%)				
Yes	32 (14.5%)	17 (11.0%)	–	–
No	188 (85.5%)	138 (89.0%)	–	–

*Note*: *pc < 0.05, significant difference (χ^2^ test).

Abbreviations: SZ, schizophrenia; HC, healthy controls; n.s, no statistical significance.

Inclusion criteria for schizophrenia patients were patients diagnosed according to the Diagnostic and Statistical Manual of Mental Disorders, 4th edition (DSM‐IV) (APA, 1994) without any age restriction. Exclusion criteria for schizophrenia patients were patients with other mental illnesses and mental illnesses caused by organic brain and physical diseases; patients with mental retardation; patients with chronic physical diseases, such as diabetes; pregnant or lactating women.

Exclusion criteria for healthy controls were positive history of mental illness and family history of mental illness; neurodevelopmental delay; existing chronic physical diseases such as diabetes; pregnant and lactating women.

### 2.2 DNA extraction

Genomic DNA was extracted from subjects' peripheral blood (EDTA anticoagulated) using standard salting‐out methods. All samples were stored in a −20°C freezer.

### 2.3 PCR‐SBT and genotyping

#### 2.3.1 Primers design

Primers MICA6823‐1F 5’‐CGTTCTTGTCCCTTTGCCCGTGTGC‐3′ and MICA9023‐4R 5’‐GATGCTGCCCCATTCCCTTCCCAA‐3′ were designed for the full‐length gene sequence of exons 2–5 of MICA gene.^30^ The amplified target DNA fragment was about 2.2 Kb. The above primers were synthesized by the Shanghai Sangon Biotechnology Company. The human growth hormone gene was used as an internal reference: an upstream primer: 5′‐GCCTTCCCAACCATTCCCTTA‐3′; downstream primer: 5′‐GAGAAAGGCCTGGAGGATTC‐3′, and the PCR product size was 834 bp. SBT sequencing primers^30^ were Exon2: 1F 5′‐ATTTCCTGCCCCAGGAAGGTTGG‐3′; Exon3: 2R5′‐CAACTCTAGCAGAATTGGAG‐3′; Exon4: 3F 5′‐AAGAGAAACAGCCCTGTTCCTCTCC‐3′; Exon5: 4R 5′‐GATGCTGCCCCATTCCCTTCCCAA‐3′.

#### 2.3.2 PCR

Reaction conditions for the PCR were 10× PCR buffer 3 μl, dNTP (2.5 mM) 3 μl, forward primer 6823 (10pM) 1.5 μl, reverse primer 9023 (10 pM) 1.5 μl, Taq enzyme (3.5 U/μl) 0.6 μl, genomic DNA (35 ng/μl) 7.5 μl, and ddH2O 12.9 μl. PCR conditions were pre‐denaturation at 95°C for 2 min; 35 cycles of denaturation at 95°C for 15 s, annealing at 66°C for 30 s, and extension at 72°C for 2 min; extension at 72°C for 10 min. Finally, the products were stored at 4°C.

#### 2.3.3 Agarose electrophoresis to identify the amplification effect

A 3 μl PCR amplification product was mixed with a 3 μl 0.5× TAE electrophoresis loading buffer. Perform constant voltage (100 V) electrophoresis was performed on 1.0% agarose. After 20 min, the electrophoretic bands were observed under the UV gel image analysis system.

#### 2.3.4 Pre‐processing of PCR amplification products for gene sequencing

After the PCR reaction, 3.6 μl of the mixture of alkaline phosphatase and exonuclease I were added to the PCR product solution before sequencing. After incubation at 37°C for 30 min, the product was placed at −80°C for 20 min to inactivate all enzyme activities.

#### 2.3.5 PCR reaction before sequencing

**FIGURE 1:**
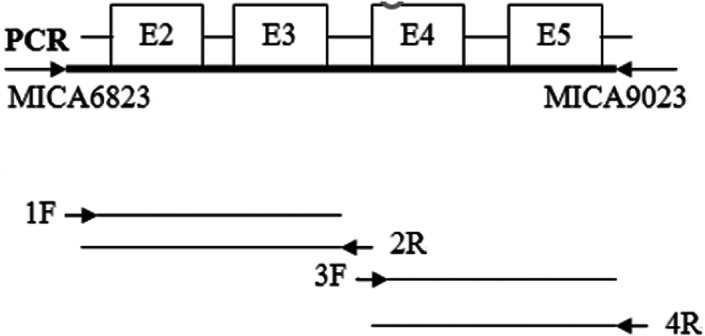
MICA genotyping and DNA sequencing strategy

The strategies for primers and gene sequencing^31^ are shown in Figure 1. Four different sequencing reactions were performed for each DNA product. The sequencing reaction system included: 3 μl of purified PCR product, 2 μl of BigDye 5× buffer, 2 μl of sequencing primer 10 pmol/μl, 1.5 μl of BigDye, and 6.5 μl of ddH2O. The total volume of the above reaction system was 15 μl. PCR conditions for the sequencing reaction were 95°C for 1 min, 96°C for 10 s, 50°C for 5 s, 60°C for 2 min, 25 cycles. Finally, the product was stored at 4°C.

#### 2.3.6 Pre‐processing for sequencing

After the PCR reaction before sequencing was completed, 1.5 μl of SDS (2%) was added to the product to denature the DNA. The steps were performed as follows: 2.5 μl of freshly prepared sodium acetate/EDTA mixture and 25 μl of absolute ethanol were added to each well in turn. After incubation in the greenhouse for 15 min, samples were centrifuged at 2500 *g* for 30 min. The Sephadex plate was then inverted and centrifuged at 250 *g* for 1 min and then spin‐dried. Then, 50 μl of freshly prepared 80% ethanol was added to each well, and samples were centrifuged at 2500 *g* for 15 min. The Sephadex plate was then inverted, centrifuged again at 250 *g* for 1 min, and then spin‐dried. Consequently, 10 μl Hi‐Di formamide was added to each well, denatured at 96°C for 3 min, centrifuged at 250 *g* for 30 s, and finally sequenced in an ABI sequencer.

#### 2.3.7 Analysis of MICA SBT sequencing results

After the DNA sequencing was completed, the instrument automatically generated 4 electronic files of DNA sequences. The segmented DNA sequences were then aligned and sheared. The two gene sequence files generated by the MICA 1F and 2R primer sequence reaction, including exon 2 and exon 3 sequences, were shown in both forward and reversed directions; the MICA 3F and 4R primer sequence reactions also produced forward and reversed two gene sequence files. Orientation of the DNA sequence file, including exon 4 and exon 5 (TM sequence). The 4R primer sequence was used for MICA‐STR (MICA exon 5 short tandem repeat) typing of the TM region.

### 2.4 Statistical analysis

MICA allele frequencies were directly calculated. The Hardy–Weinberg equilibrium test was performed with the population genetics software Arlequin v3.5. To assess the difference in allele distribution frequencies between patients with schizophrenia and healthy controls, SPSS v.23.0 (IBM, New York, USA) statistical software was used; χ^2^ test with Bonferroni correction was used for multiple comparisons and calculation of corrected probabilities (*pc*). The significance level was set as *pc* <0.05.

## 3 RESULTS

### 3.1 Hardy–Weinberg Equilibrium (HWE) test for MICA loci

There was no significant difference between the observed and theoretical frequencies of the selected subjects' genotypes (*p* > 0.05) between Han and Li schizophrenia populations and healthy controls. Also, all data were in line with the Hardy–Weinberg equilibrium law.

### 3.2 Correlation analysis of MICA allele frequency and schizophrenia in Han and Li populations in Hainan Province

#### 3.2.1 Correlation between MICA allele frequency and schizophrenia in Hainan Han population

In the study, 11 MICA alleles and 5 MICA‐STR genotypes were detected in the Han schizophrenia patients in Hainan Province. The 10 MICA alleles and 5 MICA‐STR genotypes in the Han healthy control group were identical to those in the Han schizophrenia patients **(**Tables 2 and 3 **)**.

**TABLE 2:** Association between MICA gene polymorphisms and SZ in Hainan Han nationality

*MICA* alleles	Number of alleles in SZ patients (2*n* = 440)	Allele frequency in SZ patients (%)	Number of alleles in HC (2*n* = 94)	Allele frequency in HC (%)	χ^2^	*p*	*pc*
MICA*002:01	80	18.2	5	5.3	9.575	0.002	0.024*
MICA*004	2	0.4	2	2.1	1.100	0.294	n.s
MICA*007	8	1.8	0	0	–	–	–
MICA*008	89	20.2	30	31.9	6.109	0.013	n.s
MICA*009	16	3.6	7	7.4	1.882	0.170	n.s
MICA*010	83	18.9	21	22.3	0.597	0.440	n.s
MICA*012:01	35	8	6	6.4	0.270	0.603	n.s
MICA*016	0	0	1	1.1	–	–	–
MICA*019	43	9.8	14	14.9	2.130	0.144	n.s
MICA*027	23	5.2	4	4.3	0.017	0.896	n.s
MICA*033	3	0.7	0	0	–	–	–
MICA*045	58	13.2	4	4.3	6.014	0.014	n.s

*Note*: *pc < 0.05, significant difference (χ^2^ test).

Abbreviations: SZ, schizophrenia; HC, healthy controls; n.s, no statistical significance.

**TABLE 3:** Association between MICA‐STR gene polymorphisms and SZ in Hainan Han nationality

*MICA‐STR* genotype	Number of alleles in SZ (2*n* = 440)	Allele frequency in SZ (%)	Number of alleles in HC (2*n* = 94)	Allele frequency in HC (%)	χ^2^	*p*	*pc*
MICA*A4	103	23.4	10	10.6	7.572	0.006	0.030*
MICA*A5	152	34.5	39	41.5	1.626	0.202	n.s
MICA*A5.1	89	20.2	30	31.9	6.109	0.013	n.s
MICA*A6	18	4.1	9	9.6	3.776	0.052	n.s
MICA*A9	78	17.7	6	6.4	7.519	0.006	0.031*

*Note*: *pc <0.05, significant difference (χ^2^ test).

Abbreviations: SZ, schizophrenia; HC, healthy controls; n.s, no statistical significance.

MIC A*008 had the highest allele frequency (20.2% and 31.9%, respectively) in the schizophrenia patient group and control group, followed by MICA*010 (18.9% and 22.3%, respectively). After comparing the allele frequencies of the two groups, the MICA*002:01 allele frequency (18.2%) in schizophrenia patients was significantly higher than that in the healthy control group (5.3%) (the χ^2^ value was 9.575, and the *p*‐value was 0.002). After Bonferroni correction, the *pc* value of 0.024 was still <0.05, indicating that the difference was statistically significant. The MICA*008 allele frequency (20.2%) in patients with schizophrenia was relatively lower than that in the control group (31.9%). After the χ^2^ test in the two groups, the χ^2^ value was 6.109, and the *p*‐value was 0.013 (*p* < 0.05). After correction, there was no statistical significance (*pc* = 0.156 > 0.05).

The frequency of MICA*045 (13.2%) was higher than that of the control group (4.3%) (the *p*‐value of the χ^2^ test was <0.05 [χ^2^ = 6.014, *p* = 0.014]). After correction, there was no statistical significance (*pc* = 0.168 > 0.05). In addition, this study did not detect MICA*007 in healthy controls, but this allele frequency was 1.8% in schizophrenia patients; however, due to the low value, no statistical conclusions could be drawn.

The results of MICA‐STR genotyping showed that MICA*A5 had the highest allele frequency in both the schizophrenia patient and the control groups, which were 34.5% and 41.5%, respectively, but the difference was not statistically significant. Moreover, the allele frequencies of MICA*A4 (χ^2^ = 7.572, *p* = 0.006, *pc* = 0.030) and MICA*A9 (χ^2^ = 7.519, *p* = 0.006, *pc* = 0.031) in patients with schizophrenia were all different from those in healthy controls (*p* values <0.05 after adjusting the values). Although the *p*‐value of MICA*A5.1 was <0.05 after the χ^2^ test (χ^2^ = 6.109, *p* = 0.013), the *pc* = 0.156 was >0.05.

#### 3.2.2 Correlation analysis of MICA allele frequency and schizophrenia in the Hainan Li population

A total of 10 MICA alleles and 5 MICA‐STR genotypes were detected in the Li schizophrenia population in Hainan Province. A total of 9 MICA alleles and 5 MICA‐STR genotypes were identical in schizophrenia patients and healthy controls. The MICA*A002:01 allele frequency was highest in both schizophrenia and healthy controls at 23.2% and 22.9%, respectively. The allele that was not detected in healthy controls was MICA*007, while its frequency in the schizophrenia group was 0.7%. Comparing the frequency of the MICA*010 allele in the patient group (22.9%) and the control group (11.4%), after the χ^2^ test, χ^2^ = 5.957, *p* = 0.015 < 0.05, but there was no statistical significance after Bonferroni correction (*pc* = 0.180 > 0.05) **(**Table 4 **)**.

**TABLE 4:** Association between MICA gene polymorphisms and SZ in Hainan Li nationality

*MICA* alleles	Number of alleles in SZ patients (2*n* = 310)	Allele frequency in SZ patients (%)	Number of alleles in HC (2*n* = 96)	Allele frequency in HC (%)	χ^2^	*p*	*pc*
MICA*001:01	1	0.3	2	2.1	1.163	0.281	n.s
MICA*002:01	72	23.2	22	22.9	0.004	0.950	n.s
MICA*007	2	0.7	0	0	–	–	–
MICA*008	41	13.2	13	13.5	0.006	0.937	n.s
MICA*009	2	0.7	2	2.1	0.430	0.512	n.s
MICA*010	71	22.9	11	11.4	5.957	0.015	n.s
MICA*012:01	54	17.4	18	18.8	0.089	0.766	n.s
MICA*019	14	4.5	7	7.3	0.655	0.418	n.s
MICA*027	8	2.6	3	3.1	0.000	1.000	n.s
MICA*045	45	14.5	18	18.8	1.002	0.317	n.s

*Note*: *pc < 0.05, significant difference (χ^2^ test).

Abbreviations: SZ, schizophrenia; HC, healthy controls; n.s, no statistical significance.

MICA‐STR genotyping indicted that MICA*A4 had the highest frequency in the schizophrenia population and healthy controls, with 33.2% and 35.4%, respectively, and the lowest frequency genotype in both groups was MICA*A6. After comparing genotype frequencies between the two groups, no significant differences were found **(**Table 5 **)**.

**TABLE 5:** Association between MICA‐STR gene polymorphisms and SZ in Hainan Li nationality

*MICA‐STR* genotype	Number of alleles in SZ patients (2*n* = 310)	Allele frequency in SZ patients (%)	Number of alleles in HC (2*n* = 96)	Allele frequency in HC (%)	χ^2^	*p*	*pc*
MICA*A4	103	33.2	34	35.4	0.157	0.692	n.s
MICA*A5	92	29.7	21	21.9	2.222	0.136	n.s
MICA*A5.1	41	13.2	13	13.5	0.006	0.937	n.s
MICA*A6	2	0.7	2	2.1	0.430	0.512	n.s
MICA*A9	72	23.2	26	27.1	0.596	0.440	n.s

*Note*: *pc < 0.05, significant difference (χ^2^ test).

Abbreviations: SZ, schizophrenia; HC, healthy controls; n.s, no statistical significance.

## 4 DISCUSSION

This study investigated the association between MICA gene polymorphisms and schizophrenia in Han and Li populations in Hainan Province. Even after Bonferroni correction, the allele frequencies of MICA*002:01, MICA*A4, and MICA*A9 significantly differed between Han schizophrenia patients and Han healthy controls, indicating that these alleles may be related to the susceptibility of schizophrenia in the Han population. Thus, these data imply that carrying the MICA*002:01, MICA*A4, and MICA*A9 alleles may increase schizophrenia risk. Although the correlation between MICA*007 allele frequency and schizophrenia in the Han population was not significant, it also seems to be related to schizophrenia, because it was absent in healthy controls. In the Li population, the MICA*010 allele frequency of schizophrenia patients was relatively higher than that of healthy controls, but the difference was not statistically significant after Bonferroni correction. Also, no significant differences were found for the remaining MICA alleles. We speculate that the MICA gene may increase the risk of schizophrenia by affecting immune regulation or fetal neurodevelopment. In addition, in this study, no MICA alleles significantly associated with schizophrenia in the Li population were found, which may imply that the effect of MICA on schizophrenia is associated with ethnic differences.

The frequency of MICA*008 (31.9%) in Hainan Han nationality was similar to that in the Han population from mainland (39.6%), but the frequency of MICA*002:01 (5.3%) in Hainan Han population was much lower than that in the mainland Han Chinese population (30.6%).^26^ Difference in frequency of MICA*002:01 indicated that the geographic segregation may partially contributed to the observed variations in MICA gene polymorphisms among the Hainan Han population. Regarding the allele frequency, MICA*A5 was the most common MICA‐STRs followed by MICA*A5.1, which was in line with the observations in other Han Chinese populations.^26^, ^32^, ^33^ The data of Hainan Li population were similar to Lin et al. reported before.^34^

Schizophrenia is a complex genetic disorder. Interactions of genes and their products, and interactions between genes and environmental risk factors, may increase the risk of schizophrenia.^35^ Among the schizophrenia patients enrolled in this study, only a minority had a positive family history (14.5% of Han nationality and 11.0% of Li nationality). Most relatives of patients with schizophrenia had no similar medical or family history, and schizophrenia has been demonstrated to be genetically related through twin and foster child studies.^36^ At present, Mendelian inheritance alone cannot explain the pathogenesis of schizophrenia in patients. Multiple genome‐wide association studies have identified the MHC locus (human HLA) as an important association region in schizophrenia.^18^, ^37^, ^38^ MHC has a more important role in susceptibility to schizophrenia than other psychiatric disorders.^37^ Numerous studies also provided support for the involvement of the immune system in the pathogenesis of schizophrenia; HLA class I (classical and non‐classical), class II, and class III (complement system) have been reported in schizophrenia.^19^, ^39^, ^40^, ^41^, ^42^, ^43^ Inflammation has also been identified as a risk factor for schizophrenia, possibly interacting with genetic variants in schizophrenia‐associated HLA loci to alter the risk of developing schizophrenia.^44^ MICA is a member of the non‐classical HLA class I family with the greatest degree of polymorphism.^45^ And one of two functional genes in the major histocompatibility complex class I chain‐associated gene (MIC) family. Another gene in the MIC family is MICB, which is also associated with schizophrenia risk.^46^

Soluble form of MICA can be released by human tumor cells, causing the downregulation of NKG2D, which is considered to promote tumor immune evasion and also to compromise host resistance to infections.^22^ A two‐stage case–control study shows that rs2523454 may influences susceptibility to persistent HBV infection in the Chinese population by downregulating the expression of MICA.^47^ NK cells are present in a low frequency in patients with human hepatocellular carcinoma (HCC) and their function is also impaired. MICA on the surface of HCC cells could be highly expressed by knocking out NLRP3 in HCC, which led to the effective NK cytotoxicity.^48^ MICA gene variations are associated with risk of immune‐mediated disease, a meta‐analysis shows that the MICA‐TM A9 allele is associated with psoriasis susceptibility in Asian populations and that the MICA‐TM A9 allele is associated with a psoriatic arthritis risk in Europeans.^49^

This study has a few limitations. First, it has a relatively small samples size. However, the selection of research subjects was strict; the enrolled patients did not suffer from any other abnormal physical diseases that could cause bias, and the selected subjects were from a strictly homogeneous ethnicity. Second, this study only discussed schizophrenia from the MICA gene polymorphism, which has certain limitations. MICA alleles may be just a part of haplotype or reflection of linkage disequilibrium, the real effect on schizophrenia may be through HLA. It would be interesting to which HLA alleles the most important MICA alleles correspond.

The strength of this article is that it simultaneously studied two ethnicities and that the effect of racial differences on the findings was taken into account. Also, there is no research report on the relationship between MICA and schizophrenia. In our future studies, we plan to investigate the effect of overexpression of MICA on cell proliferation and apoptosis through corresponding cell function experiments and explore the pathophysiological relationship between MICA and schizophrenia. Meanwhile, we need to expand the sample size and use multi‐ethnic studies to fully describe the impact of MICA on schizophrenia to verify and evaluate whether and how MICA is associated with schizophrenia. Moreover, further research will reveal the link between genotype and animal phenotype by establishing gene mutation animal models. Also, further studies are needed to establish whether the MICA gene mutation is widespread in the schizophrenia population and to determine its exact pathogenesis. Elucidating the etiology and specific pathogenesis of schizophrenia will help prevent and treat schizophrenia, which in turn will reduce the burden of disease treatment.

## 5 CONCLUSIONS

We found MICA*002:01, MICA*A4, and MICA*A9 may be susceptibility alleles for schizophrenia in the Han population, while the MICA allele polymorphism in the Li population is not associated with schizophrenia in Chinese.

## CONFLICT OF INTEREST

All authors declare no competing interests.

## FUNDING INFORMATION

The study was supported by the Finance Science and Technology Project of Hainan Province, Grant/Award Number: ZDYF2022SHFZ2952, ZDYF(XGFY)2020002 and ZDYF2018132.

## Data Availability

The datasets used and/or analyzed during the current study are available from the corresponding author on reasonable request.
